# A missense mutation in *MYH1* is associated with susceptibility to immune-mediated myositis in Quarter Horses

**DOI:** 10.1186/s13395-018-0155-0

**Published:** 2018-03-06

**Authors:** Carrie J. Finno, Giuliana Gianino, Sudeep Perumbakkam, Zoë J. Williams, Matthew H. Bordbari, Keri L. Gardner, Erin Burns, Sichong Peng, Sian A. Durward-Akhurst, Stephanie J. Valberg

**Affiliations:** 1 Department of Population Health and Reproduction, University of California, Davis SVM, Room 4206 Vet Med 3A, One Shields Ave, Davis, CA 95616 USA; 2 Department of Large Animal Clinical Sciences, Michigan State University, East Lansing, MI 48824 USA; 3 Department of Veterinary Population Medicine, College of Veterinary Medicine, University of Minnesota, St. Paul, MN 55108 USA

**Keywords:** Equine, Genome-wide association, Immunology, Myopathy, Myosin heavy chain 1

## Abstract

**Background:**

The cause of immune-mediated myositis (IMM), characterized by recurrent, rapid-onset muscle atrophy in Quarter Horses (QH), is unknown. The histopathologic hallmark of IMM is lymphocytic infiltration of myofibers. The purpose of this study was to identify putative functional variants associated with equine IMM.

**Methods:**

A genome-wide association (GWA) study was performed on 36 IMM QHs and 54 breed matched unaffected QHs from the same environment using the Equine SNP50 and SNP70 genotyping arrays.

**Results:**

A mixed model analysis identified nine SNPs within a ~ 2.87 Mb region on chr11 that were significantly (*P*_unadjusted_ < 1.4 × 10^− 6^) associated with the IMM phenotype. Associated haplotypes within this region encompassed 38 annotated genes, including four myosin genes (*MYH1*, *MYH2*, *MYH3*, and *MYH13*). Whole genome sequencing of four IMM and four unaffected QHs identified a single segregating nonsynonymous E321G mutation in *MYH1* encoding myosin heavy chain 2X. Genotyping of additional 35 IMM and 22 unaffected QHs confirmed an association (*P* = 2.9 × 10^− 5^), and the putative mutation was absent in 175 horses from 21 non-QH breeds. Lymphocytic infiltrates occurred in type 2X myofibers and the proportion of 2X fibers was decreased in the presence of inflammation. Protein modeling and contact/stability analysis identified 14 residues affected by the mutation which significantly decreased stability.

**Conclusions:**

We conclude that a mutation in *MYH1* is highly associated with susceptibility to the IMM phenotype in QH-related breeds. This is the first report of a mutation in *MYH1* and the first link between a skeletal muscle myosin mutation and autoimmune disease.

**Electronic supplementary material:**

The online version of this article (10.1186/s13395-018-0155-0) contains supplementary material, which is available to authorized users.

## Background

Inflammatory myopathies are infectious or immune-mediated disorders that are characterized by the presence of lymphocytes in the skeletal muscle. Immune**-**mediated myositides (IMMs) are an important cause of morbidity and, in some cases, mortality in several species including humans [1], dogs [2], and horses [3, 4]. Common clinical features include malaise, muscle atrophy**,** and weakness with a histopathologic hallmark of inflammatory infiltrates, particularly lymphocytes, surrounding blood vessels**,** and within myocytes [5, 6]. There are several different IMM subtypes including inclusion body myositis in humans [7], polymyositis and dermatomyositis in dogs and humans [5], canine masticatory myositis [8]**,** and equine IMM [3, 4]. Equine IMM is characterized by CD4+, CD8+**,** and CD20+ lymphocytic infiltrates surrounding blood vessels and infiltrating myofibers without evidence of rimmed vacuoles [3, 6]. Similar to human IMM, equine IMM has a bimodal age distribution affecting young horses (< 8 years of age) or older horses (> 17 years of age) [3, 9].

Causes of autoimmune diseases such as IMM are not well understood, but environmental stimuli, combined with a genetic predilection, appear to be important initiating factors [10, 11].The precise environmental trigger for equine IMM is not clear, but 39% of horses with IMM are reported to have a recent history of infection, particularly with S*treptococci* spp., or vaccination with influenza, herpes virus-1**,** or *Streptococcus equi subsp. equi 3* to 4 weeks prior to onset [3, 4]. While recurrence of muscle wasting is reported with equine IMM, an improvement in clinical signs is often noted following treatment with corticosteroids. Full muscle mass is typically regained in 1–10 weeks, with corticosteroid treatment decreasing the time to full recovery [3].

Genetic associations with IMM have been found with various major histocompatibility complex loci in humans and dogs [10, 12]. Because the majority of horses affected by IMM are of Quarter Horse (QH)-related breeds and since certain stallions appear to be overrepresented in the genetic lineage of QHs with IMM, we hypothesized that there is an underlying genetic variant that causes susceptibility to IMM in QHs [3, 13]. The first objective of this study was to identify associated genomic regions underlying risk for developing equine IMM by performing a genome-wide association (GWA) study of QHs and related breeds with and without IMM that were housed in the same environment and therefore exposed to the same risk factors that may result in the IMM phenotype. The second objective was to evaluate the region of association from the GWA using whole genome sequencing to identify a putative functional variant associated with the equine IMM phenotype. The third objective was to determine if the alteration encoded by the putative functional variant altered protein structure and was targeted by inflammation.

## Methods

All blood and muscle samples were collected with approval from the Animal Care and Use Committee at the University of Minnesota and University of California, Davis.

### IMM case and control selection

#### GWA cohort

**Fig. 1:**
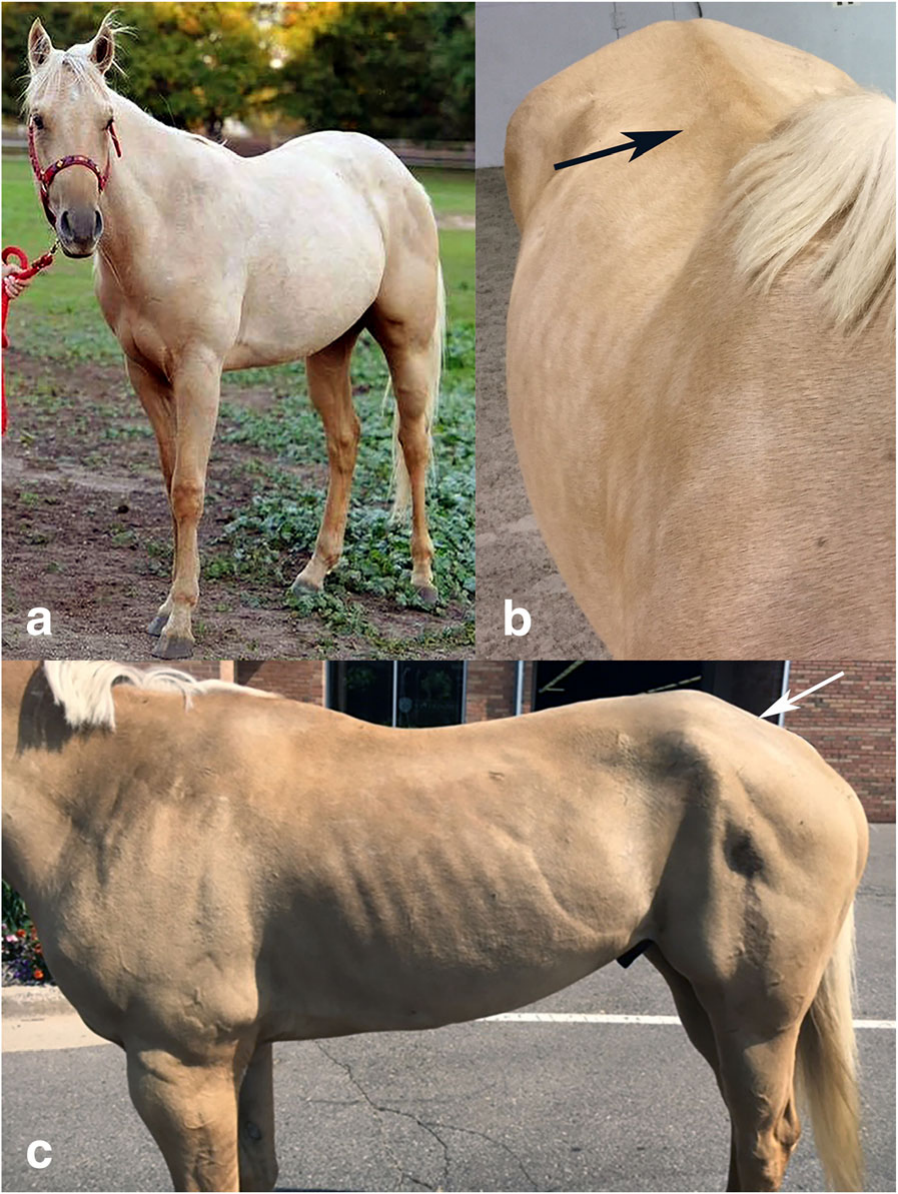
**a** Normal muscle mass in an *MYH1* E321G homozygote prior to developing IMM. **b** The same horse 4 months after an episode of IMM. The spine is prominent due to loss of epaxial muscles (arrow). **c** Atrophy of middle and superficial gluteal muscles (arrow) is present

IMM-affected QHs (*n* = 36; 11 geldings, 10 stallions, and 15 mares) were selected based on a history of muscle atrophy (particularly of the epaxial or gluteal muscles, Fig. 1) and the presence of lymphocytes invading myofibers or cuffing blood vessels in a formalin-fixed or fresh muscle biopsy as previously described [3]. Horses with type 1 polysaccharide storage myopathy based on amylase-resistant polysaccharide in myofibers or the presence of the H309A *GYS1* mutation [14] were excluded. The mean ± SEM age at the time of biopsy was 4.6 ± 0.8 years (range 0.1–19 years) for IMM-affected QHs. Due to the importance of environmental triggers, unaffected QHs (*n* = 54; 5.6 ± 0.6 years range 1–17; 17 geldings, 3 stallions and 34 mares) were selected from two herds that had active IMM cases. Unaffected horses had no history of muscle atrophy or stiffness consistent with IMM. Horses were selected such that they were not related at least within one generation. Of the horses used for the GWA, 1/36 affected and 41/54 unaffected horses were used in a previous genetic study [15].

#### Whole-genome sequencing

From the GWA cohort, four of the most severely affected IMM QHs (1 gelding, 1 stallion, and 2 mares) and four unaffected QHs (2 geldings and 2 mares) were selected for whole-genome sequencing.

#### Follow-up cohort

The follow-up cohort included an additional 35 IMM QHs (13 geldings, 11 stallions, 11 mares; mean age at biopsy 3.4 ± 0.8 years [range 0.5–18 years]), phenotyped clinically by muscle atrophy (Fig. 1) (*n* = 25/35) and stiffness (*n* = 4/35) with mild lymphocytic infiltrates in myocytes or vascular cuffing in muscle samples. Unaffected QHs (*n* = 22; 5 geldings, 2 stallions, 15 mares, > 2 years) in this follow-up cohort were housed on the same property as IMM-affected cases and therefore exposed to the same environmental risk factors to develop IMM. While these horses may have gone on to develop myositis later in life, all had no history of disease at the time of sampling. All horses in this cohort were genotyped for the non-synonymous *MYH1* E321G variant.

#### Random QH cohort

A cohort of 28 healthy QHs (*n* = 22) and a related breed, Paint horses (6), that were embryo transfer recipient horses of unknown bloodlines were genotyped for the putative variant to assess the prevalence of the *MYH1* E321G variant in distantly related or unrelated cohorts. There was no history of IMM in this herd.

#### Across breed cohort

Genotyping of the *MYH1* E321G variant was performed in a total of 64 horses across 6 breeds (Additional file 1: Table S1). Additionally, publically mapped whole-genome sequences from the Sequence Read Archive (SRA; https:/www.ncbi.nlm.nih.gov/sra) were available for 110 horses across 21 breeds.

### Pedigree analysis

From the original GWA cohort, pedigrees were available for 23/36 QH. Pedigrees were analyzed using Pedigraph [16]. Following genotyping of all horses for the *MYH1* E321G variant, additional pedigrees were created of individual families.

### Genome-wide association (GWA)

DNA isolations were prepared from whole blood (ArchivePure™ DNA Blood Kit VWR International, Radnor, PA) or muscle (ArchivePure™ DNA Tissue Kit VWR International, Radnor, PA) samples according to the provided protocols. Genotyping of a subset of samples was performed on the Equine SNP 50K BeadChip (Illumina, San Diego, CA) (1 IMM and 41 unaffected horses), prior to the creation of the Equine SNP 70K BeadChip (Illumina, San Diego, CA). Thirty-five IMM QHs and 13 unaffected QHs were genotyped across 74,500 SNP markers with the Equine SNP 70K BeadChip (Illumina, San Diego, CA).

#### Statistical methods and data analysis

Datasets were merged and only SNPs that passed quality control settings (minor allele frequency > 1%, genotyping across individuals > 90%, and Hardy-Weinberg *p* > 0.001) were selected. A genome-wide efficient mixed model association was performed using GEMMA software using the standardized relatedness matrix option (−gk 2) [17]. Population stratification was estimated by assessing the genomic control inflation factor (*λ*). A Bonferroni correction for 39,589 tests (the number of useable SNPs) from the GEMMA analysis, based on a *P*_genome-wide_ of 0.05, was determined as 1.26 × 10^− 6^.

### Haplotype analysis

For chr11, which demonstrated the only genome-wide significant associations on the GWA, haplotypes were reconstructed on the individual chromosome using Haploview [18]. SNPs were filtered based on genotyping (> 90%) and minor allele frequency (> 1%). Association testing of both the single markers and haplotypes was performed using 1000 permutations. The adjusted haplotype-wide significance threshold was *P*_permuted_ = 0.05.

### Whole-genome sequencing

Using Illumina’s TruSeq DNA PCR-free library preparation kit (Illumina, San Diego, CA, USA) and following the manufacturer’s instructions, libraries were prepared with median insert size of 300–400 bp from the four IMM QHs and four unaffected QHs. The eight libraries were barcoded and pooled across eight lanes of a 125PE flow cell on an Illumina HiSeq2500, generating an average of 10.2× coverage per horse. Following quality trimming, reads were mapped to the EquCab2.0 reference genome using the Burrows-Wheeler Aligner (BWA) version 0.7.5a [19] using default settings. After sorting the mapped reads by the coordinates of the sequence, PCR duplicates were labeled with Picard tools (http://sourceforge.net/projects/picard/). The Genome Analysis Tool Kit (GATK version v.2.7.4) was used to perform local realignment [20]. Variant calls were made across all eight samples simultaneously using standard hard filtering parameters or variant quality score recalibration with Haplotype Caller according to GATK Best Practices Recommendations [21, 22].

#### Statistical methods and data analysis

SnpEFF [23] and SnpSift [24] were used to predict the functional effects of detected variants across the genome and within chr11 candidate region and filter by segregation using Fisher’s exact test. Variants were filtered by region in the entire associated haplotype block on chr11:49,915,548–56,207,873. To further refine the segregation, an unaffected Arabian horse from a previously published study [25] was included. Within this region, segregating variants were further filtered by the Fisher’s exact allelic *P* value (< 0.0003), allowing for one heterozygote in the IMM-affected group. Segregating variants within this region were further evaluated using all publically available mapped whole-genome sequences in the NCBI Sequence Read Archive (https://www.ncbi.nlm.nih.gov/sra). Putative segregating variants were excluded if found in > 1 breed other than the QH. In addition to variant calling, visual inspection of the raw reads using the Integrated Genomics Viewer [26] within the chr11 region of association was performed. As conservation scores are not available in the EquCab2.0 genome browser within UCSC (https://genome.ucsc.edu/), scores were determined for each orthologous human variant using the 100 vertebrate score by phastCons (https://genome.ucsc.edu/). Whole genome sequences were deposited in the NCBI Sequence Read Archive (https://submit.ncbi.nlm.nih.gov/subs/sra/) (SRP119975).

### Genotyping

Primer pairs were designed using Primer3Plus software [27] (F-CCCAAGATCTCAATGGCACT and R-ACCTTGTGGGAACATTCAGC) to amplify and subsequently genotype the nonsynonymous *MYH1* E321G variant in an additional cohort of IMM-affected and unaffected QHs and a cohort of unaffected Arabian horses. Amplification of products was performed using endpoint PCR and visualized with the QIAxcel Advance System (QIAGEN, Valencia, CA, USA) and the QIAxcel DNA Screening Kit (QIAGEN, Valencia, CA, USA). The 20-μL PCR reactions were comprised of 2 U of Hot-start TAQ and 2.0 μL of 10× Buffer (Applied Biosystems, Foster City, CA, USA), 0.25 mM dNTPs (Thermo Fisher, Waltham, MA, USA), 0.5 μM of both forward and reverse primers (Invitrogen Life Technologies, Carlsbad, CA, USA), and 20 ng genomic DNA. Standard PCR conditions were performed as follows: hot-start TAQ activation and initial denaturation at 95 °C for 10 min; 35 cycles of 95 °C denaturation for 30 s, 60 °C annealing for 1 min, and 72 °C extension for 1 min; and final extension at 72 °C for 10 min. PCR products were purified using ExoSAP-IT® PCR Product Cleanup Kit (Affymetrix, San Diego, CA, USA). Sanger sequencing was performed using ABI 2500 automated sequencers. Resulting sequences were aligned to EquCab2.0 (http://www.ncbi.nlm.nih.gov/genome/145) and analyzed with Sequencher® software (Gene Codes Corporation, Ann Arbor, MI, USA).

### Quantitative real-time PCR

Skeletal muscle *MYH1* expression was assessed in 14 IMM-affected QH (11 E321G *MYH1* homozygotes, 3 heterozygotes) and 11 unaffected horses across various breeds (all homozygous wild-type). Primers were designed for *MYH1* (F-CACCACCAACCCGTATGACT and R-GAAGCCCAAGATCTCAATGG) and the reference gene *ACTB* (F-AAGGAGAAGCTCTGCTATGTCG and R-GGGCAGCTCGTAGCTCTTC), RT-qPCR was performed, and data were analyzed as previously reported [28].

### Protein modeling and side chain analysis

Conformational changes caused by the identified E321G *MYH1* variant were modeled using online G23D tool [29] and *Homo sapiens* myosin gene chain A (PDB ID: 4pa0). The amino acid in the mutated position was modeled using SCcomp [30]. Contact surface areas and solvent accessible surface areas were calculated using G23D, which applies Voronoi tessellation to allocate contact surfaces between neighboring atoms [31, 32]. Stability analysis of the *E317G* variant was performed using I-Mutant-2 [33], directly accessed from G23D.

### Inflammation and muscle fiber type composition

Formalin-fixed or fresh skeletal muscle biopsy specimens were obtained by referring veterinarians and shipped on gel packs to the Neuromuscular Diagnostic Laboratory. Fresh samples were frozen in isopentane suspended in liquid nitrogen within 48 of the initial biopsy, and samples were stored at − 80 °C.

In a study of IMM published previously, inflammatory cell types were identified in fresh muscle samples obtained from horses of Quarter Horse-related breeds that presented with a history of gross atrophy of gluteal and epaxial muscles evident on physical examination [13]. Macrophages were identified with acid phosphatase stains, and immunohistochemical staining was used to identify CD4+, CD8+, and CD20+ [6]. Detailed methods can be found elsewhere [3, 6]. For the purposes of the present study, the type of inflammatory cells infiltrating myofibers was re-assessed in relationship to *MYH1* genotype (13 horses GG, 3 horses G/A).

Adequate well-preserved frozen muscle tissue remained after examining inflammatory cell types from 10 IMM horses that were then used to evaluate fiber types. In six IMM cases, inflammation was identified in a formalin-fixed gluteal sample and fiber typing was performed in a concomitantly submitted fresh semimembranosus sample lacking substantial inflammation. Semimembranosus or gluteal muscle samples from five horses that were homozygous wild-type for the *MYH1* E321G variant were used as unaffected controls. Unaffected horses had normal muscle mass as assessed by physical examination and lacked lymphocytes or macrophages in muscle biopsies. Frozen samples from semimembranosus (4 E321G *MYH1* homozygotes, 3 heterozygous, 4 wild-type) and middle gluteal (3 E321G *MYH1* homozygotes, 0 heterozygous, 1 wild-type) muscles were assessed.

Serial 10-μm sections stained with hematoxylin and eosin (HE) and labeled by immunofluorescence for fiber type were used to identify inflammatory infiltrates within fiber types. Type 1, 2A, 2AX, and 2X muscle fiber types were identified by multiple fluorescent labeling according to Tulloch et al. [34]. Briefly, sections were incubated with a goat polyclonal anti-collagen V IgG antibody (1350-01 Southern Biotech, Birmingham, AL) 1:100 for 1 h at room temperature. Next, three separate mouse monoclonal antibodies to detect type 1, slow myosin IgG 1:100 (MAB1628 Millipore, Burlington, MA), type 2a IgG 1:6 (A4.74 DSHB), and both type 2A and 2X IgG 1:10 (NCL-MHCf Leica Biosystems, Buffalo Grove, IL) were conjugated to fluorescent IgG_1_ Fab fragments using Zenon® Mouse IgG labeling kits (Life Technologies, Carlsbad, CA), Alexa Fluor® 488 (A4.74), Alexa Fluor® 594 (NCL-MHCF), and Pacific Blue™ (MAB1628). The three Zenon® labeled antibodies were mixed together, added to the tissue sections, and incubated at 4 °C overnight. A secondary antibody for Collagen V, FITC-rabbit anti-goat IgG (61-1611, Invitrogen, Carlsbad, CA) 1:500 was applied to the cryosections and incubated for 1 h at room temperature. Sections were subsequently mounted using VECTASHIELD mounting medium (H1000, Vector Labs, Burlingame, CA) and examined using a fluorescence microscope (Olympus, Tokyo, Japan) with filters designed for each of the different emitting wavelengths. Images were captured and pseudo-colored composites generated.

#### Statistical methods and data analysis

The total number of type 1, 2A, 2AX, and 2X muscle fibers was determined for the entire muscle section, and fiber type compositions were determined by dividing the total number of fibers of each type by the total number of muscle fibers counted (range 447 to 3244 muscle fibers/sample). The fiber type composition of IMM samples with inflammation and wild-type samples were compared by genotype and disease status using a two-way ANOVA.

## Results

### Pedigree analysis

From the original GWA cohort, pedigrees were available for 23/36 QH. All affected horses could be traced back to a common sire within eight generations (Additional file 2: Figure S1). Pedigree analysis supported either an autosomal dominant or autosomal recessive mode of inheritance.

### Genome-wide association study

**Fig. 2:**
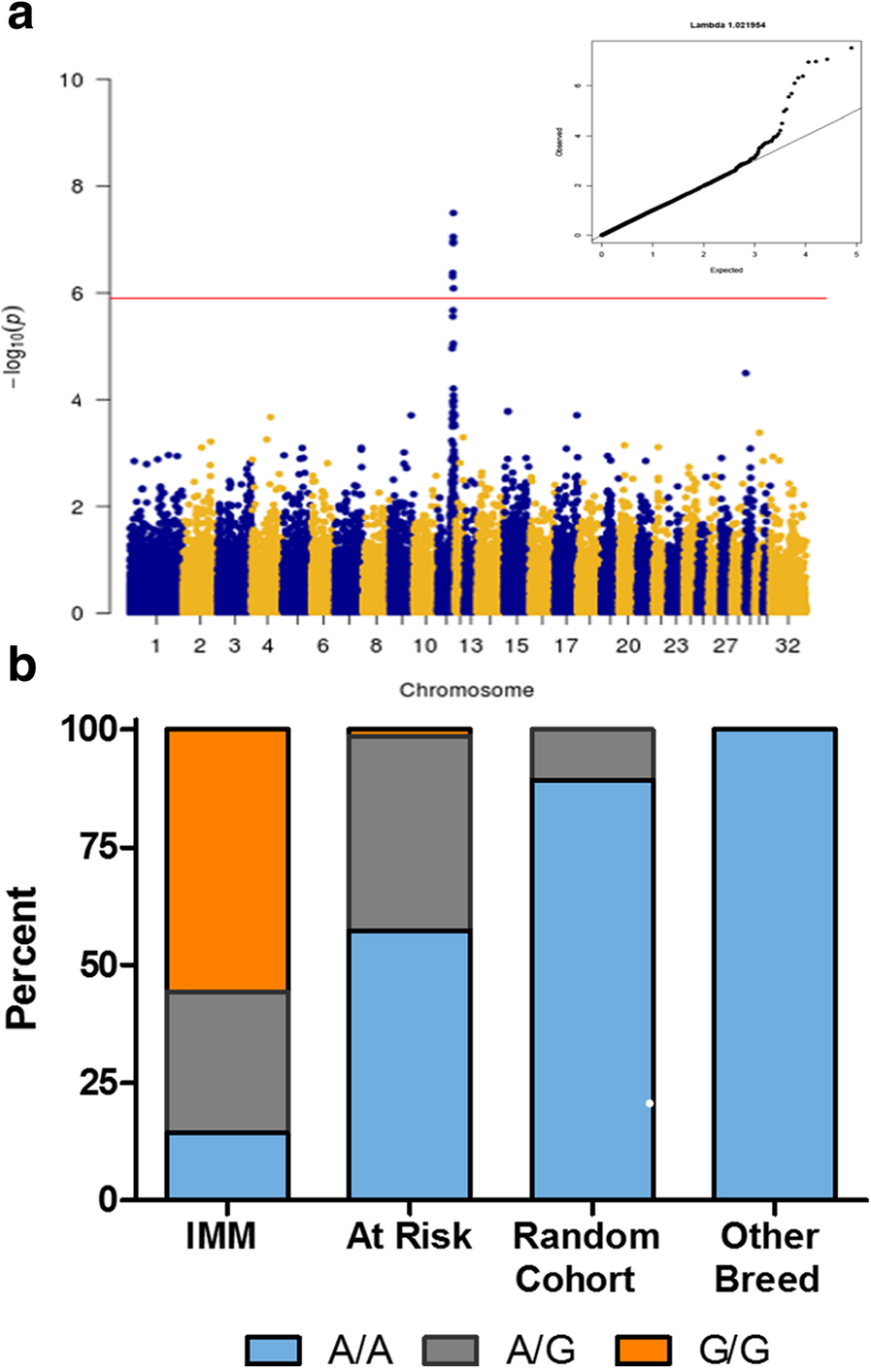
**a** Manhattan plot and (top right insert) QQ-plot demonstrating a genome-wide significant association with the IMM phenotype using GEMMA analysis [17] on chr11. Minimal genomic inflation was present. **b** Genotypes for the *MYH1* E321G variant across IMM-affected horses (*n* = 71 GWA and follow-up cohorts combined), at risk horses (*n* = 75, housed on the same farms as IMM horses), a cohort of random QH (*n* = 28), and 21 other breeds (*n* = 179)

Following quality control of the 73,706 SNPs available on the array, 39,589 SNPs remained (1601 excluded for minor allele frequency < 1%, 32,439 excluded for genotyping < 90%, and 77 excluded for failing hardy Weinberg equilibrium [*P* < 0.001]). Genomic inflation (*λ*) was estimated at 1.98, indicative of population stratification. Due to the elevated genomic inflation, a mixed model analysis was performed utilizing GEMMA with the same filters [17]. Using the GEMMA relationship matrix, genomic inflation was controlled for in the population (*λ* = 1.02). Nine SNPs on chromosome 11 reached genome-wide significance (Table 1 and Fig. 2a).

**Table 1:** Genome-wide significant single nucleotide (SNP) polymorphisms for immune-mediated myositis (IMM)

Chr	Position	Ref	Alt	p_score
11	54549083	A	G	1.52055E−08
11	53982070	C	T	4.0689E−08
11	53911268	A	G	4.53797E−08
11	53889957	G	A	4.78016E−08
11	52379255	A	G	1.53355E−07
11	51677777	G	A	2.28682E−07
11	54437293	A	G	5.70386E−07
11	52382557	T	G	8.81654E−07
11	53554776	A	G	9.80409E−07

*Chr* chromosome, *Ref* reference allele, *Alt* alternative allele

### Haplotype analysis and candidate region

Haplotype analysis of 1109 SNPs on chr11 that passed quality control (838 removed for genotyping < 90% and 64 removed for minor allele frequency < 1%) using Haploview [18] identified 46 significantly associated haplotype blocks (*P*_permuted_ < 0.05), spanning ~ 6.3 Mb from chr11: 49,915,548–56,207,873. Of these, four highly significant haplotype blocks (*P*_permuted_ < 0.001) spanning ~ 3.1 Mb from chr11:52379156-55487290 were identified. This region overlapped the ~ 2.87 Mb region flanked by eight of the nine genome-wide significant SNPs from the GWA. Therefore, while the entire 6.3 Mb region was evaluated, the 3.1 Mb region was prioritized.

The 6.3 Mb region identified through GWA and subsequent haplotype analysis encompassed 148 Ensembl annotated genes, with 277 annotated transcripts in equine skeletal muscle [35]. Within the 3.1 Mb prioritized region, 38 Ensembl annotated genes and 48 annotated skeletal muscle transcripts were identified [35]. Of these 38 genes, four myosin genes (*MYH1*, *MYH2*, *MYH3*, and *MYH13*) and myocardin (*MYOCD*) were located within the associated region. *MYH1* and *MYH2* are expressed in adult equine skeletal muscle while *MYH3* is expressed at the embryonic stage and *MYH13* is expressed primarily in ocular skeletal muscle [36].

### Whole-genome sequencing

Whole-genome sequencing was performed on four IMM and four unaffected QHs at ~ 10× coverage. Visual inspection of the raw reads using the Integrated Genomics Viewer [26] within the GWA region did not identify any structural variants across the eight horses. Using Haplotype Caller according to GATK Best Practices Recommendations [21, 22], an average of 5,107,127 single nucleotide variants (SNVs) and 655,690 insertions/deletions were identified across all eight horses. Variants were filtered based on a region of interest from the GWA and haplotype analysis, spanning the entire 6.3 Mb associated region (chr11:49,915,548–56,207,873), and 628 variants within this filtered region were significantly associated with the IMM phenotype (Fisher’s exact test, *P*_unadjusted_ < 0.00003). These 628 variants were examined in all publically available mapped whole-genome sequences (*n* = 110 across 21 breeds) from the Sequence Read Archive (SRA; https://www.ncbi.nlm.nih.gov/sra). Only 15 of these variants were unique to the QH. As IMM has not been reported in a breed other than QH-related breeds, the other 613 variants were excluded from further analysis. The remaining 15 breed-specific variants were prioritized for further evaluation (Table 2). Two of these variants were classified as MODERATE missense variants while other variants were classified as MODIFIERS (*n* = 12) or a LOW synonymous variant (*n* = 1) (Table 2).

**Table 2:** Segregating variants for IMM

Variant	Ref allele	Alternate allele	SNPEff	Skeletal muscle expression [35]	Conservation score
chr11:50,342,189	G	A	Upstream, *ACAP1*	Non-coding	0.149
chr11:50,534,985	A	AG	Intergenic	Coding	0
chr11:51,060,046	G	A	Upstream, *TMEM107*	Non-coding	N/A
chr11:51,248,313	T	C	Synonymous, *KRBA2*	Coding	0
chr11:51,282,298	C	T	Intergenic	Non-coding	0
chr11:51,514,034	C	T	Intergenic	Non-coding	0.022
chr11:51,515,343	C	T	Intergenic	Non-coding	1
chr11:51,516,129	C	G	Intergenic	Non-coding	0.006
chr11:51,543,197	T	C	Intergenic	Non-coding	0
***chr11:52,944,928***	***G***	***C***	***Intergenic***	***Non-coding***	***N/A***
***chr11:52,993,878***	***T***	***C***	***Missense, MYH1***	***Coding***	***1***
***chr11:53,105,764***	***C***	***T***	***Missense, MYH3***	***Non-coding***	***1***
**chr11:53,502,949**	**G**	**GT**	**Intergenic**	**Non-coding**	**0**
chr11:53,795,669	G	A	Intronic, novel gene (ENSECAG00000025157)	Non-coding	N/A
chr11:53,830,181	C	T	Intronic, novel gene (ENSECAG00000025157)	Non-coding	N/A

The bold entries are the prioritized region based on significance of haplotype association testing. The italic entries are genotyped in additional cohort

To prioritize these 15 variants for further genotyping, the custom equine transcriptome recently published by our laboratory was utilized [35]. These RNA-Seq datasets included skeletal muscle and embryo (both inner cellular mass and trophoectoderm). Within all available sets, the *MYH3* variant was non-coding (Table 2). Therefore, the *MYH1* variant was initially prioritized for genotyping of a larger group of horses.

### Genotyping of putative variants

DNA from 46/48 horses used in the GWA study was available for genotyping the *MYH1* E321G variant and a significant association was identified (*P* = 6.76 × 10^− 15^). Within the follow-up cohort of 35 mildly affected IMM horses, a significant allelic association was validated (*P* = 2.95 × 10^− 5^) with the *MYH1* E321G variant. In the two IMM cohorts combined, 87.7% of horses were homozygous or heterozygous for the variant (Fig. 2b). In the random cohort of QHs without a history of IMM, three heterozygotes (11%) were identified. Genotyping of 175 horses across 21 non-QH breeds did not identify any other breeds with the *MYH1* E321G variant (Fig. 2b).

To further exclude the possibility that additional segregating variants were responsible for IMM, we genotyped a subset of horses for chr11:52,944,928 and chr11:53,105,764 in horses that lacked the *MYH1* E321G variant but were classified as IMM based on lymphocytic infiltrates (*n* = 2 in GWA and *n* = 7 in follow-up cohort). The subset of horses genotyped included the *n* = 9 IMM-affected horses that genotyped A/A at chr11:52,993,878, IMM-affected horses that genotyped G/G (*n* = 11), and unaffected horses that genotyped A/A (*n* = 11). The nine IMM-affected horses that had genotyped A/A at chr11:52,993,878 also genotyped for the reference allele at the two other segregating variants, resembling the unaffected horse genotype. These nine horses with an inflammatory myopathy therefore appeared to be phenocopies. In the other IMM-affected horses, the two other variants are genotyped as alternate allele/alternate allele. Because these variants lacked a strong functional effect, were non-coding in adult equine skeletal muscle, and lacked any known connection to immune or muscle function, the *MYH1* E321G variant was identified as the putative functional variant for equine IMM (Table 2).

### Pedigree evaluation based on *MYH1* E321G genotype

**Fig. 3:**
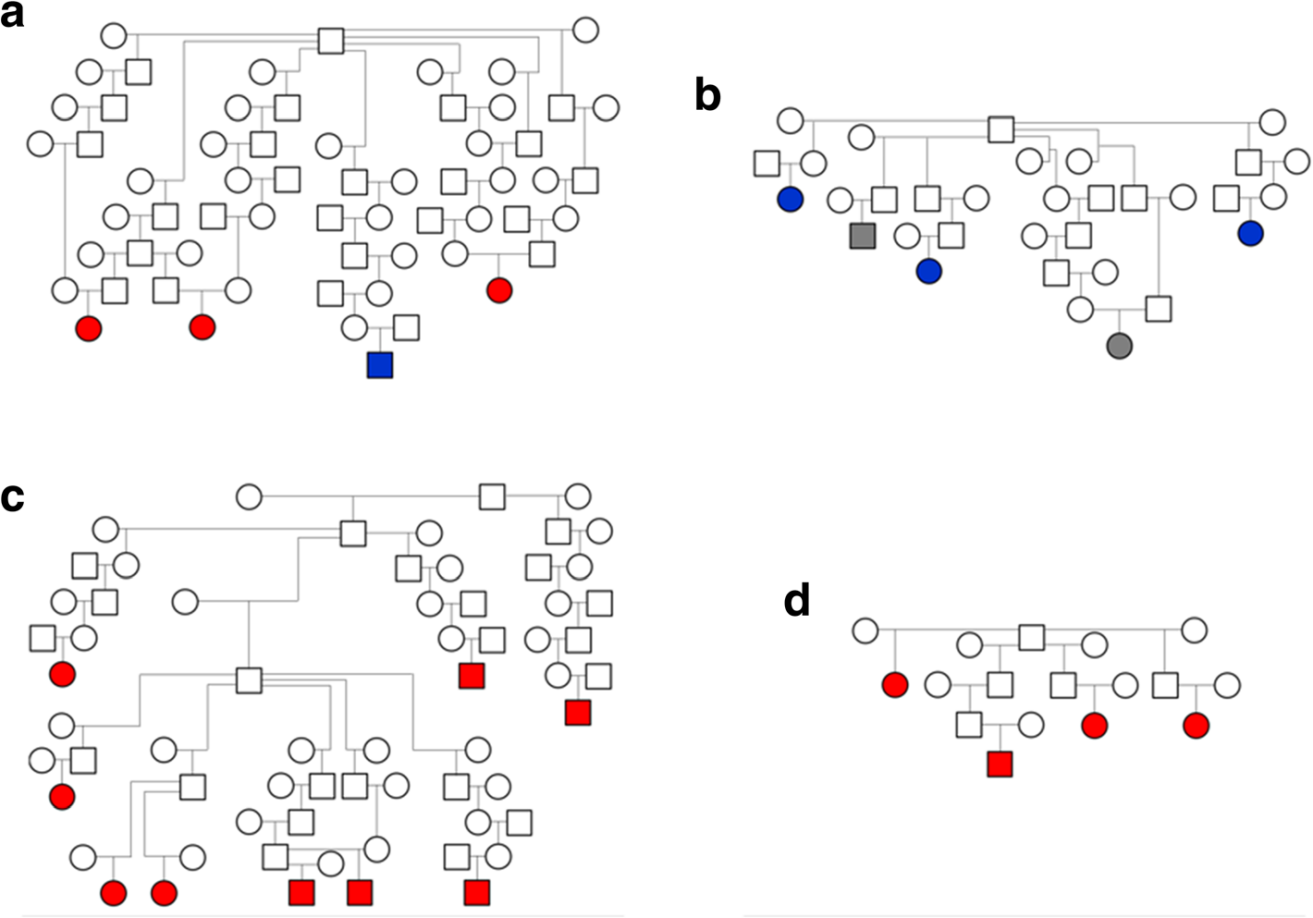
**a–d** Pedigrees from four subsets of the larger pedigree (Additional file 2: Figure S1) demonstrating a founder effect of four stallions for the IMM phenotype. Circles = females, squares = males. Open circles = phenotype unknown. Colored circles = IMM-affected with red = G/G genotype, blue = G/A genotype, and gray = A/A phenotype. Two of these founder stallions were traced back to the stallion in family A within one to three generations. For family D, the dam line traced back to the stallion in family A within four generations (Additional file 2: Figure S1)

Available pedigrees were re-evaluated on the 23 IMM-affected horses based on the *MYH1* E321G genotype and could be distinctly summarized as linking to four founder stallions (Fig. 3). Two of these founder stallions were traced back to the stallion in family A within one to three generations. For family D, the dam line traced back to the stallion in family A within four generations.

### RT-qPCR

There was no significant difference in *MYH1* skeletal muscle expression between IMM-affected horses and controls (data not shown).

### Protein modeling, contact area, and stability analysis

**Fig. 4:**
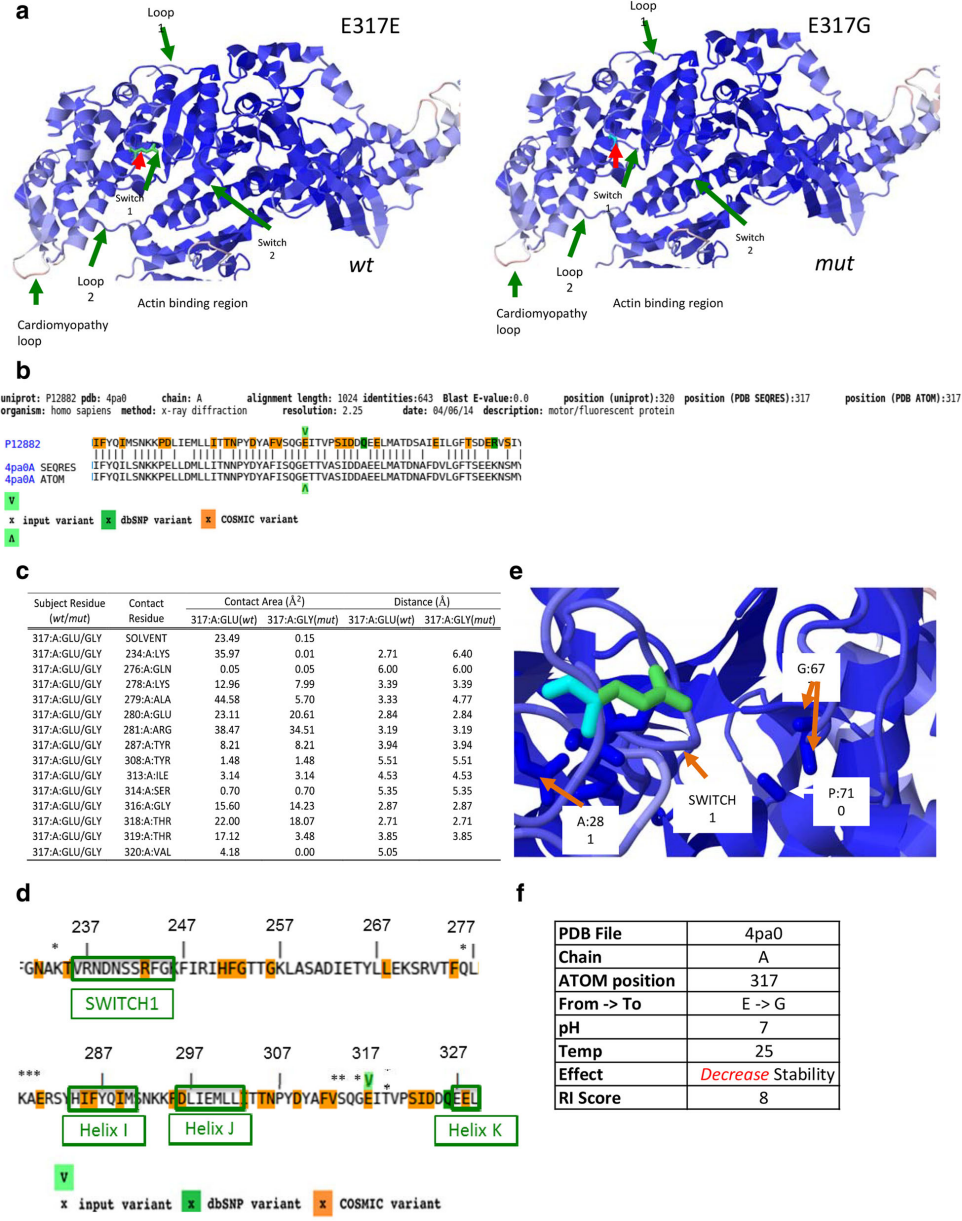
Schematic representation of MYH1 (MyHC 2X) with wild type (E317E, *wt*) and *MYH1* mutation (E317G, *mut*). **a** Wild-type (*wt*) variant is shown as light green (red arrow) and *E317G* mutation (*mut*) is shown on the adjacent panel as teal (red arrow). In both panels, helices are represented as blue and additionally loop, SWITCH regions and actin binding sites are labeled. **b** Detailed alignments of the query protein (upper sequence line) and structure hits showing the protein sequence (PDB SEQRES record) and the resolved coordinates (PDB ATOM record). The input variant is indicated by green arrows (below and above the alignment) and highlighted amino acids represent residues that appear in dbSNP as well as common associated somatic mutation in cancer (COSMIC) variants. **c** Side chain and stability analysis of *MYH1* E317G mutation showing contact surface areas between atoms and solvent accessible surfaces. Table includes contact surface area (Å^2^) and minimal atomic distance (Å) between two atoms of the two residues for the 15 amino acid residues that were determined to be impacted by the mutation. **d** A pictorial presentation of contact/residues affected by the *MYH1* E317G mutation and its association to SWITCH1, helix I and helix J regions of MYH1. The residues affected are represented with an asterisk (*). Helix K is partially represented. The input variant is indicated by green arrows (below and above the alignment) and highlighted amino acids represent residues that appear in dbSNP as well as common associated somatic mutation in cancer (COSMIC) variants. **e** G23D model displaying a close up of combined *MYH1* mutation (teal only) and wild type (green). Blue represent the helices and shows the proximity of the *MYH1* mutation to the SWITCH 1 region and to the G677 and P710 residues found between Loop2 and SH2 helix domain of MYH1. **f** Stability analysis of the E317G mutation using I-mutant-2 at physiological pH, which predicts decreased stability of the mutant protein

The missense mutation identified in *MYH1* is located in a highly conserved region of the myosin globular head in subfragment-1 between the helix loop-helix region of Helix J and Helix K (Fig. 4a, b). The helix loops lay between SWITCH1 and SWITCH2 motifs that have been identified as RAS GTP proteins, and play a role in the binding of ATP [29]. The *MYH1* E321G mutation substitutes a negatively charged glutamic acid (E) for a non-polar glycine (G) that lacks side chains necessary for hydrogen bond formation. Contact area and solute accessibility analyses showed that 14 residues were directly affected by the *MYH1* E321G mutation (Fig. 4c) with the largest reduction of contact between the SWITCH1 and helix 1 domains of the myosin 2X globular head (Fig. 4d, e). Furthermore, this single mutation of E317G is responsible for significantly decreasing the stability of the protein at physiological conditions with a RI score of 8 (Fig. 4f).

### Inflammation and muscle fiber type composition

**Fig. 5:**
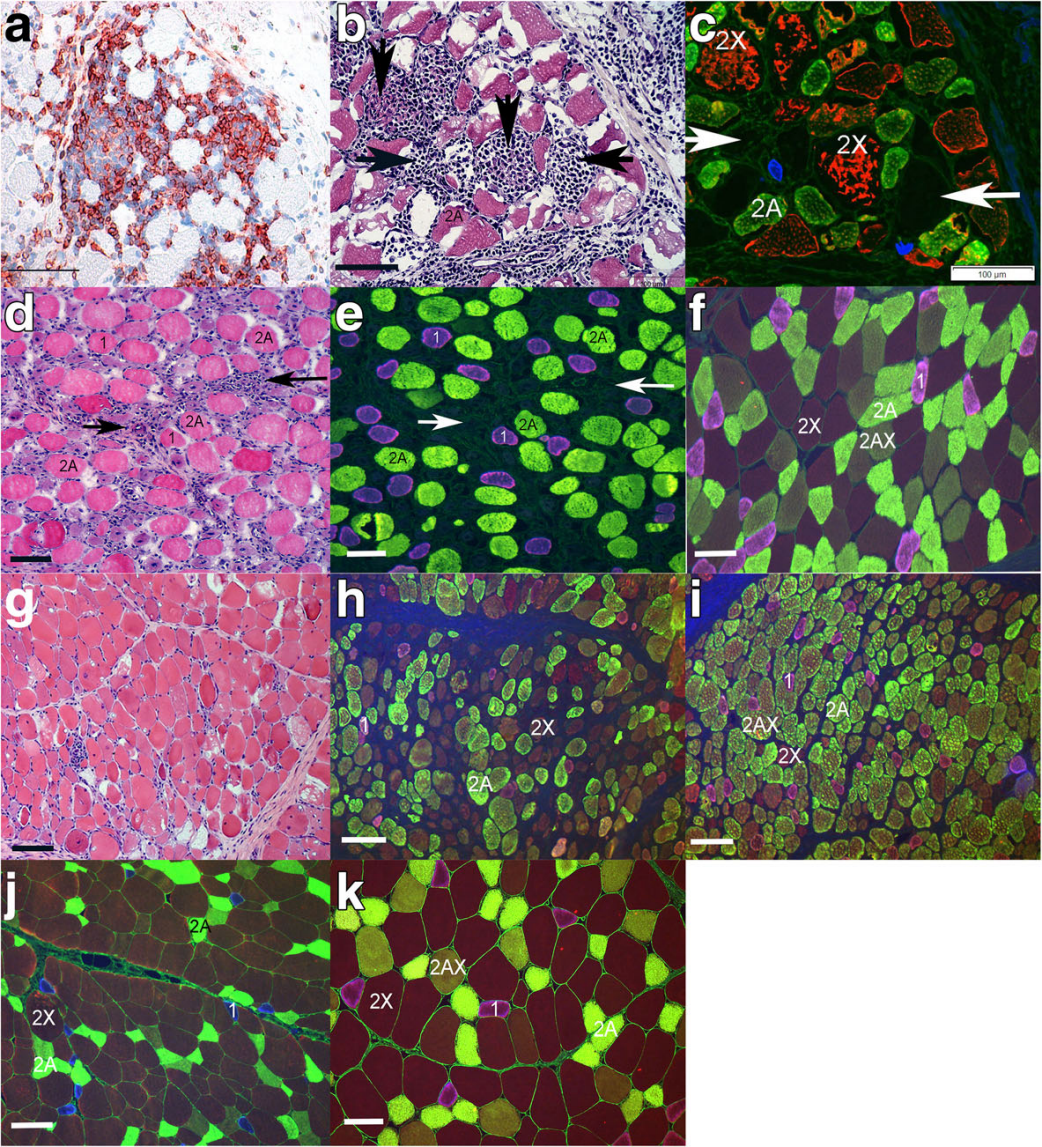
**a** Marked CD4+ lymphocytic infiltrates (red) within myofibers of horse 1, an E321G *MYH1* homozygote. IHC × 20. **b** Serial section to **c** demonstrating inflammatory cell infiltrates (arrows) in horse 1. HE × 20. **c** Serial section to **b** demonstrating that fibers infiltrated by lymphocytes (vertical arrows) are 2X fibers (red) and that some fibers with cellular infiltrates (horizontal arrows) do not have any remaining myosin staining. IF × 20. **d** Serial section to **e** from horse 2 showing inflammatory cells (arrows). HE × 10. **e** Serial section to D demonstrating a complete absence of 2X fibers. Dark regions with no staining (arrows) correspond to regions of inflammation in D. IF × 10. **f** Normal fiber type distribution and fiber sizes in another homozygous wild-type horse for comparison. IF × 20. **g** Section of muscle from horse 3 that had a 4-month history of IMM showing a small amount of inflammatory cell infiltrates. HE × 10. **h** Another section of horse 3 demonstrating atrophic type 2X fibers (brown). IF × 10. **i** Preponderance of type 2A (yellow) or 2AX fibers (intermediate) over type 2X fibers (brown) in another region of the sample from horse 3. IF × 10. **j** Normal fiber type distribution of muscle from a wild-type horse (type 1 blue, type 2A yellow, type 2X brown) showing type 2X fibers that are larger than type 2A fibers. IF × 10. **k** Normal fiber type distribution and fiber sizes in the semimembranosus muscle that lacks inflammation from horse 4 that was homozygous for the E321G variant. IF × 20. Bars in all figures represents 100 μm

CD4+ and CD8+ lymphocytes and macrophages were identified within and surrounding myofibers in all horses homozygous and heterozygous for the E321G *MYH1* variant (Fig. 5a). Inflammatory cells were present in type 2X fibers which contain myosin heavy chain 2X that is encoded by *MYH1* (Fig. 5b, c). Significantly fewer type 2X fibers (mean 27%; range 0–50%) were found in E321G *MYH1* homozygous and heterozygous samples with inflammation compared to controls (mean 60; range 43–87%; *P* < 0.0001) (Fig. 5d, e) Small numbers of inflammatory cells, atrophic type 2X fibers, and a predominance of type 2A and 2AX fibers over 2X fibers were identified in the semimembranosus muscle of an IMM homozygote with a 4-month long history of IMM (Fig. 5f–h). In contrast, in the complete absence of inflammation, the proportion of type 1, 2A, 2AX, and 2X fibers of control horses did not differ significantly from IMM muscle samples (Fig. 5i–k). The mean percentage of type 2X fibers in IMM E321G horse muscle without inflammatory infiltrates (4 semimembranosus, 2 gluteal) was 45%, ranging from 30 to 56%, as compared to control samples (3 semimembranosus, 2 gluteal), where the mean percentage of type 2X fibers was 60%, ranging from 43–87%.

## Discussion

This is the first report of a mutation in *MYH1* gene associated with susceptibility to a specific myopathy. A GWA study initially identified a ~ 6.3 Mb region on chr11 associated with IMM, and haplotype analyses narrowed this region to ~ 3.1 Mb that encompassed two myosin genes expressed in adult equine muscle, namely *MYH1*, encoding type 2X myosin heavy chain (MyHC), and *MYH2*, encoding MyHC 2A. Type 2X fibers comprise 40–80% of all fibers and type 2A comprise 30-40% of all fibers present in muscles typically affected with IMM [37]. One nonsynonymous variant was identified in *MYH1* that resulted in substitution of a charged glutamic acid for a non-polar amino acid, glycine, at 321 in the MyHC globular head. Credence for the significance of the variant was provided by the fact that glutamic acid is highly conserved (Table 2), present in this position in seven other species (Sus, Bos, Homo, Oryctol, Rat, Canis) and in the corresponding position of MyHC 2A, perinatal, extraocular, embryonic, and cardiac/slow MyHC [38]. We propose that the other segregating variants with no known functional effect were in linkage disequilibrium with this *MYH1* functional variant, although they cannot be conclusively excluded as putative variants for IMM. The *MYH1* E321G variant is all the more significant, however, because of a strong selection pressure against mutations in skeletal muscle. MyHC appears to exist based on the remarkable orthology of myosin heavy chain genes across species [38, 39]. Thus, the E321G variant identified in *MYH1* in IMM horses is both novel and strongly associated with susceptibility to develop equine IMM.

The *MYH1* E321G variant appears to be variably penetrant, conferring susceptibility to disease, potentially dependent upon whether other factors needed to trigger an immune-mediated disease are present. A significant proportion of both IMM and in contact unaffected horses were heterozygous for the *MYH1* E321G variant. Heterozygosity, however, was much less in the unrelated group of QH and absent in the Arabian breed of horse as well as in the publically available mapped NCBI SRA database of 21 breeds. The high degree of heterozygosity in the original cohort of horses can be explained by the fact that the group of horses in contact with IMM-affected horses were closely related to the affected horses. The unadjusted genomic inflation factor for the initial GWA was 1.98. To control for environmental exposure to factors such as other respiratory bacteria, viruses, and vaccinations that can trigger IMM, we selected horses in the same environment, which was a breeding farm in one case and a private farm with preference for certain bloodlines in another [4]. The M protein of *S. equi* in particular shows considerable similarity in amino acid sequence with MyHC 2X (encoded by *MYH1*; Additional file 3: Figure S2) and is a common trigger of IMM [3]. Lack of IMM onset in some heterozygotes could be the result of differential expression of the affected allele, subthreshold environmental triggering of autoimmunity, or a lack of initial priming of the immune system from a first exposure to mutant MyHC 2X. It is also possible that some of the in contact horses developed IMM at a later stage. Together, our results suggest that rather than consistently causing a myopathy, homozygosity and, in some cases, heterozygosity for the *MYH1* variant predisposed horses to a myopathy under certain environmental triggers.

Nine of 71 horses diagnosed with IMM and genotyped for *MYH1* E321G were homozygous reference. Of these, two were from the initial GWA cohort, where horses were phenotyped more stringently (i.e., moderate to severe lymphocytic infiltrate on muscle biopsy), and seven were from the follow-up cohort, where IMM-affected cases had a milder degree of myofiber lymphocytic infiltrates and included those with only perivascular lymphocytes. As additional nearby variants segregated with the IMM phenotype at identical frequencies as *MYH1* E321G (Table 2), these nine horses were genotyped for these additional variants. All nine horses genotyped homozygous for the reference allele at the two additional variants, identical to the population of unaffected horses. Lymphocytic infiltrates in the skeletal muscle are not specific for immune-mediated myositis and can be found in other inflammatory myopathies [2, 13] with sarcocystosis being the most common equine infectious myopathy characterized by lymphocytic infiltrates [40, 41]. The nine horses lacking the *MYH1* E321G variant could very well represent phenocopies due to an inflammatory myopathy. Phenocopies were to be expected because differentiation of immune-mediated versus inflammatory myopathies of infectious origin is difficult in horses based solely on muscle histopathology [2, 13, 42].

In total, 15 variants segregated at identical frequencies with the IMM phenotype (Table 2). While most of these variants were classified as having LOW or MODIFIER effects by SNPEff [23], two were characterized as having MODERATE effects (missense variants in *MYH3* and *MYH1*) when using the Ensembl annotation. We have recently demonstrated that this annotation is not highly accurate in the horse, and we have published a tissue-specific transcriptome for the horse based on RNA-sequencing data and integration with other EquCab2.0 annotations, including NCBI [35]. This custom annotation includes transcriptomes of equine skeletal muscle and embryo. Across all tissues, the *MYH3* variant was identified as non-coding. Therefore, we propose that these variants are in linkage disequilibrium with the *MYH1* E321G variant.

Mutations in *MYH2* have been reported in a small number of human patients. Recessive truncating deletions in *MYH2* result in early-onset weakness confined to extraocular, semitendinosus, gracilis, vastus lateralis, and medial gastrocnemius muscles whereas dominant point mutations in *MYH2* result in a later onset of mild progressive weakness [43–45]. Both recessive and dominant *MYH2* mutations result in mild myopathic changes such as variability of fiber size, internalized nuclei, and increased interstitial connective and adipose tissue; however, only recessive deletions produce a total absence of type 2A myofibers [43, 45]. Unlike patients with recessive *MYH2* mutations, in horses with IMM, the reduction in type 2X myofibers was dependent upon inflammation and, in the absence of inflammation, homozygous IMM horses had a normal proportion of type 2X fibers. In the small number of samples evaluated from homozygous horses with chronic atrophy, we found a decrease in the proportion of type 2X myofibers with a higher proportion of type 2A and 2AX fibers. Our results suggest that lymphocytic destruction of MyHC 2X fibers appears to be a prerequisite for acute inflammation in IMM horses. Previous studies have not identified immunoglobulin bound to myofibers in IMM horses [3]; however, circulating anti-*MYH1* myosin or cytokines have not been studied in equine IMM. The clinical signs and muscle histopathology of IMM appear to be distinct from those previously reported for *MYH2* mutations in other species.

In contrast to the low frequency of mutations in *MYH1* and *MYH2*, more than 500 disease-causing point mutations have been described in *MYH7*, with the majority producing hypertrophic or dilated cardiomyopathy [46, 47]. A minority of *MYH7* mutations are reported to cause skeletal myopathies such as myosin storage myopathies or Laing distal myopathy [46, 47]. One family with a p.K1729del in *MYH7* had similar inflammatory changes to those seen in equine IMM, although the clinical presentation was that of distal limb weakness not the rapid proximal muscle atrophy seen in equine IMM [48]. Similar to equine IMM, increased skeletal muscle MHC class I expression and perivascular and endomysial lymphocytic infiltrates (CD3^+^, CD4^+^, and CD8^+^) were found in this family with Laing distal myopathy, along with rimmed vacuoles, which are not a feature of equine IMM [6, 48]. Laing distal myopathy has highly variable muscle pathology, however, and inflammation is not a consistent feature of the skeletal muscle in most patients [49].

The best characterized link between myosin, inflammation, and muscle disease would be immune-mediated myocarditis [50]. Fragments of cardiac myosin have been shown to activate Toll-like receptors (TLR2), which strongly drive reactivity to self and subsequently determine the type of adaptive immune response (i.e., Th1, Th2) that occurs [51]. Synergy between the activated innate immune response and the adaptive response of pathogenic T cell epitopes appear to be important in the generation of chronic myocardial inflammation [51]. Similar to human myocarditis, the adaptive immune response could be triggered in IMM horses by shared epitopes between bacteria such as the M protein of group A *Streptococcus sp.* and myosin (Additional file 3: Figure S2) [52]. The innate immune response could be triggered in IMM by release of the mutant form of MyHC 2X from myofibers following muscle damage (trauma, vaccination). The loss of hydrogen bonds with the *MYH1* mutation could possibly lead to conformational changes in myosin that activate TLRs and autoimmunity. Of note, a nonsynonymous mutations in *MYH7* (S545A) in DBA/2 mice appears to predispose these mice to immune-mediated myocarditis [53, 54]. When DBA/2 mice with the *MYH7* S545A variant and BALB/c mice without the variant are auto-inoculated with cardiac myosin, chronic myocarditis only occurs when serum from the DBA/2 strain is injected into DBA/2 not BALB/c mice [53]. The authors concluded that susceptibility to autoimmune myocarditis was dependent not only on the activation of self-reactive lymphocytes but also on genetically determined target organ sensitivity. Because both the DBA/2 *MYH7* mutation and the equine *MYH1* variant are located in the globular head of myosin, it is possible that a mutation in this highly conserved region somehow confers target host susceptibility to myositis.

Perhaps the most intriguing link between myosin and autoimmunity comes from canine masticatory muscle myositis (CMM). CMM presents with painful swelling followed by rapid atrophy of masticatory muscles [8]. Similar to equine IMM, biopsies of CMM masseter muscle are characterized by MHC I upregulation, B cells, and a predominance of CD4+ over CD8+ T lymphocytic infiltrates in masticatory muscles [2, 6, 8, 53]. Both within the masseter muscle and in the circulation, autoantibodies for masticatory muscle myosin (2M) or myosin binding protein-C are evident with CMM, suggesting that myosin isoforms unique to masseter muscles have antigenic potential and can serve as target antigens for inflammatory cells [55]. Genetic analysis of CMM dogs has yet to be performed to assess the potential for putative mutations in masticatory muscle myosin encoding genes to enhance susceptible to CMM.

## Conclusions

In conclusion, an E321G *MYH1* mutation is highly associated with susceptibility to IMM in horses. In the absence of inflammation, type 2X muscle fiber type composition is within normal limits in IMM horses; however, within a particular environment, the *MYH1* mutation results in invasion and destruction of type 2X myofibers by lymphocytes and rapid onset of gross muscle atrophy.

## Additional files

Additional file 1: **Table S1.** Across breed genotyping. (XLSX 9 kb)

Additional file 2: **Figure S1.** Within the cohort of genome-wide association horses affected with IMM, 23/36 had available pedigree information. All affected horses (red) could be traced back to a common sire within eight generations. Pedigree analysis supported either an autosomal dominant or autosomal recessive mode of inheritance. Circles = females, squares = males. (TIFF 7954 kb)

Additional file 3: **Figure S2.** Protein alignment of *MYH1* gene and M protein of *Streptococcus equi* (*S. equi*) (AHI46575.1) showing similarities in regions of the *S. equi* alignment and the *MYH1* gene. Sequences were aligned using CLUSTALX (version 2). (TIFF 15425 kb)

## Abbreviations

GWA: Genome-wide association
HE: Hematoxylin and eosin
IMM: Immune-mediated myositis
MHC: Major histocompatibility complex
MYH: Myosin heavy chain
QH: Quarter Horse
SNP: Single nucleotide polymorphism
WGS: Whole genome sequencing
