# Supplementary figures and images for: A missense mutation in MYH1 is associated with susceptibility to immune-mediated myositis in Quarter Horses
Source: Skelet Muscle. 2018 Mar 6;8:7. doi: 10.1186/s13395-018-0155-0 (PMC5838957; doi:10.1186/s13395-018-0155-0)

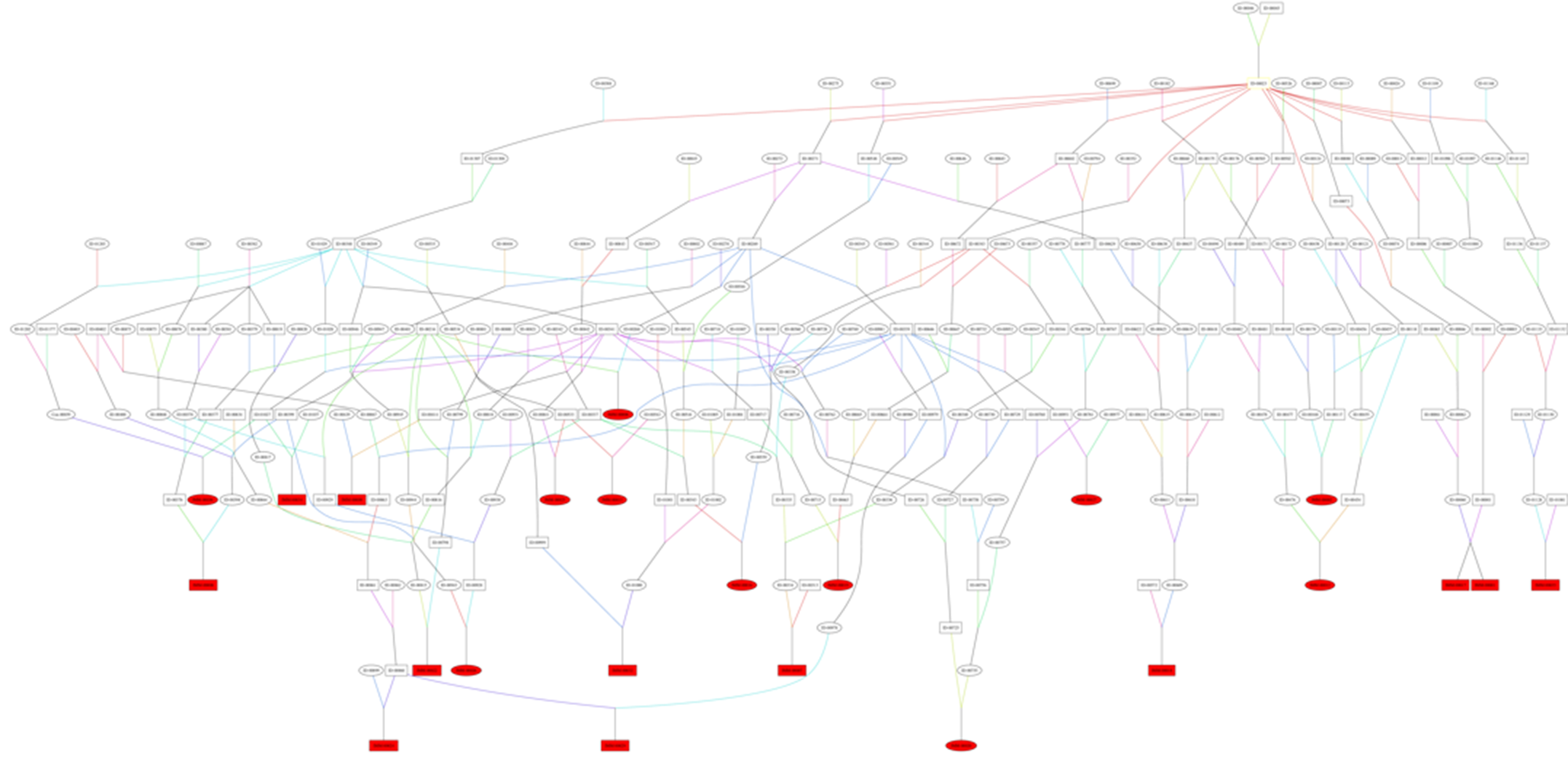

Supplement: Supplementary file 2 — Figure S1. Within the cohort of genome-wide association horses affected with IMM, 23/36 had available pedigree information. All affected horses (red) could be traced back to a common sire within eight generations. Pedigree analysis supported either an autosomal dominant or autosomal recessive mode of inheritance. Circles = females, squares = males. (TIFF 7954 kb) [file 13395_2018_155_MOESM2_ESM.tif]

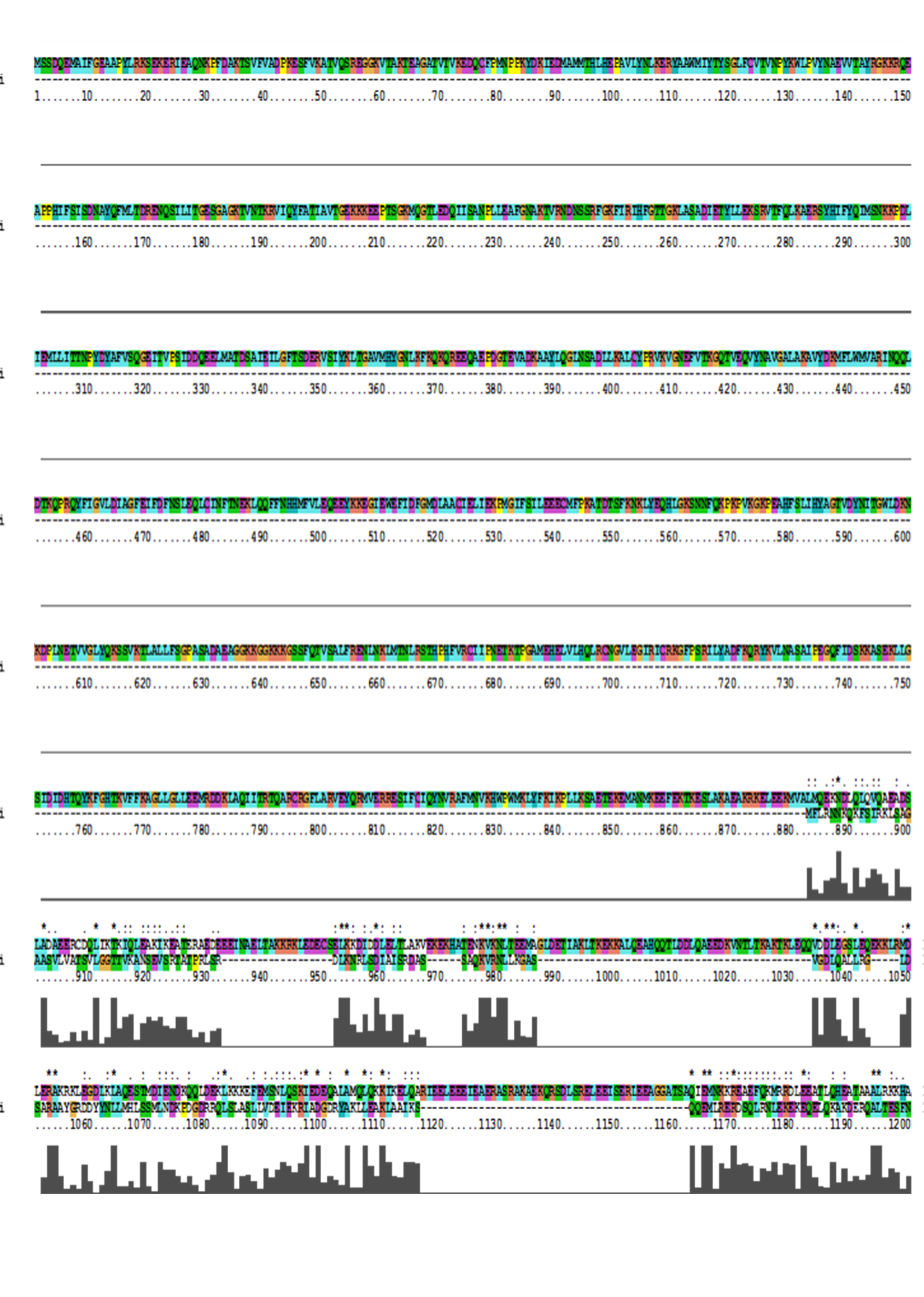

Supplement: Supplementary file 3 — Figure S2. Protein alignment of MYH1 gene and M protein of Streptococcus equi (S. equi) (AHI46575.1) showing similarities in regions of the S. equi alignment and the MYH1 gene. Sequences were aligned using CLUSTALX (version 2). (TIFF 15425 kb) [file 13395_2018_155_MOESM3_ESM.tif]
